# Hematoma formation after basivertebral nerve ablation

**DOI:** 10.1016/j.inpm.2025.100617

**Published:** 2025-07-30

**Authors:** Sean Fox, Joshua Levin

**Affiliations:** a Department of Orthopaedic Surgery, Stanford University, USA; b Department of Neurosurgery, Stanford University, USA

## Abstract

Basivertebral nerve ablation (BVNA) has been shown to have significant and lasting effects on chronic vertebrogenic low back pain. Serious complications have been rare, including hematoma formation. However, given that the target lesion occurs at the basivertebral foramen, which not only houses the basivertebral nerve terminus but also the basivertebral vessels, hematoma formation is theoretically plausible. This case demonstrates a suspected hematoma in the extradural neural axis compartment following BVNA. The patient was treated conservatively and there were no significant lasting adverse effects.

## 1 Introduction

Radiofrequency ablation of the basivertebral nerve (BVNA) is a relatively new treatment for chronic vertebrogenic low back pain. This condition, recognized by type 1 (bone marrow edema and hypervascularity) or type 2 (fatty replacement of red bone marrow) Modic changes (MC) on MRI is associated with chronic low back pain (CLBP) [1].

BVNA has been shown to have significant and lasting effects on vertebrogenic CLBP. Studies have shown modest to robust improvement in pain and disability in comparison to placebo and to usual care [2,3]. Long-term relief has been demonstrated, with ODI improvement from 43 to 17 (from “severe” to “minimal” disability) and a ≥50 % reduction in pain in 66 % of patients, ≥75 % reduction in pain in 47 % of patients, and complete resolution in 34 % of patients at 5-year follow-up [4].

To date, reported serious complications from BVNA have been rare. Some studies found no serious adverse events reported over a two-year period post procedure [5]. Others have reported infrequent complications including vertebral compression fracture, nerve root injury, lumbar radiculopathy, retroperitoneal hemorrhage, and transient motor or sensory deficits, all occurring at a rate of less than 2 % [2]. However, given that research and clinical trials involving BVNA has occurred primarily in the last decade, additional adverse events may be observed as more procedures are performed. Given that BVNA involves passing a cannula and radiofrequency transducer in close proximity to blood vessels within the vertebral body, one potential risk from the procedure is hematoma.

## 2 Case report

A 72-year-old man with Parkinson's disease presented with chronic, mostly axial low back pain, possibly exacerbated by lumbar extension, with less pain into the right and occasionally left posterior thigh. MRI showed type 1 Modic changes at L5-S1 and degenerative canal stenosis at L4-5. A right L5-S1 transforaminal epidural steroid injection was performed, resulting in very temporary 90 % relief of pain. Next, bilateral L3, L4 medial branch and L5 dorsal ramus blocks were performed, with negative diagnostic results. At this point, his pain was suspected to be primarily vertebrogenic in nature, and L5, S1 BVNA was performed. The patient stopped taking his aspirin 81mg seven days prior to the procedure and was taking no other anticoagulants. He had no other medical comorbidities such as chronic kidney disease or the use of medications such as selective serotonin reuptake inhibitors associated with platelet dysfunction.

**Fig. 1:**
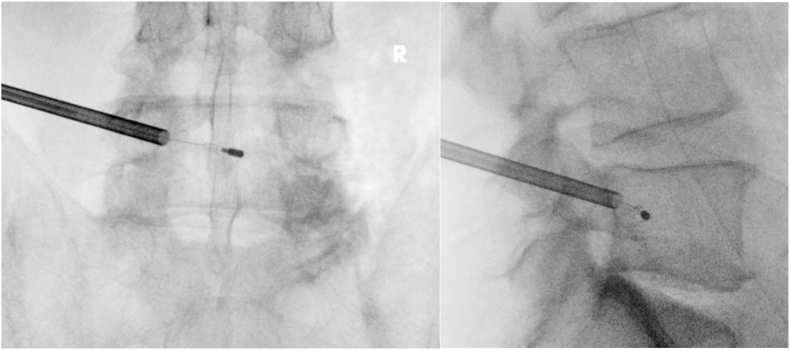
L5 BNVA procedure images.

**Fig. 2:**
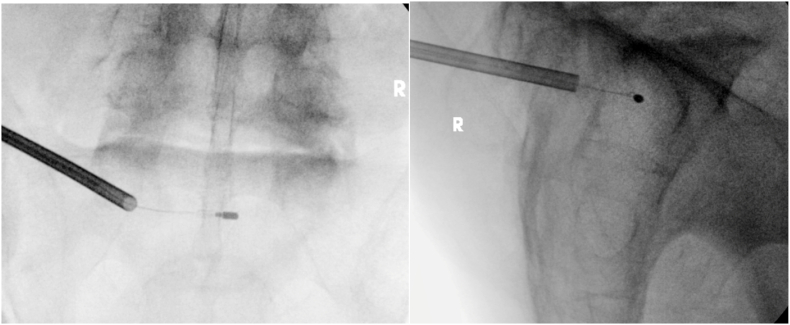
S1 BVNA procedure images.

The BVNA was carried out via the standard posterolateral approach to the L5 and S1 pedicles. The L5 ablation was performed successfully from a left-sided approach (Fig. 1). The procedure at S1 was started via a right-sided approach, however the stylet was unable to be advanced to midline while remaining adequately posterior. Thereafter, a second attempt was performed successfully at S1 from a left-sided approach (Fig. 2). There were no immediate complications from the procedure, and the patient ambulated out of the surgery center.

Two weeks later, he reported 90 % improvement in symptoms with residual soreness after the procedure. At subsequent follow-up three months post-procedure, he reported continued relief, although only 50 % improved from his prior baseline. His back pain at this time was in the same location. At follow-up three months later (six months post-procedure) his pain had regressed to the pre-procedure baseline, though now localized axially with no symptoms into the legs. He remained neurologically intact without any deficits. A new lumbar spine MRI was obtained eight months post-procedure which revealed well-positioned L5 and S1 basivertebral nerve ablation lesions and a new finding of increased T2 signal posterior to the L5 vertebral body with tenting of the posterior longitudinal ligament, without significant stenosis. This finding was suspected to represent a hematoma. The case was discussed with surgical colleagues, and given the absence of neurologic deficits, observation was pursued. Three months later, the patient reported some improvement in his pain that was difficult to quantify, but a definite improvement. A subsequent MRI was obtained (11 months post-procedure), and this demonstrated persistence of the hematoma but improvement in its size (See Fig. 3A, Figure 3B, Fig. 3C).

**Fig. 3A:**
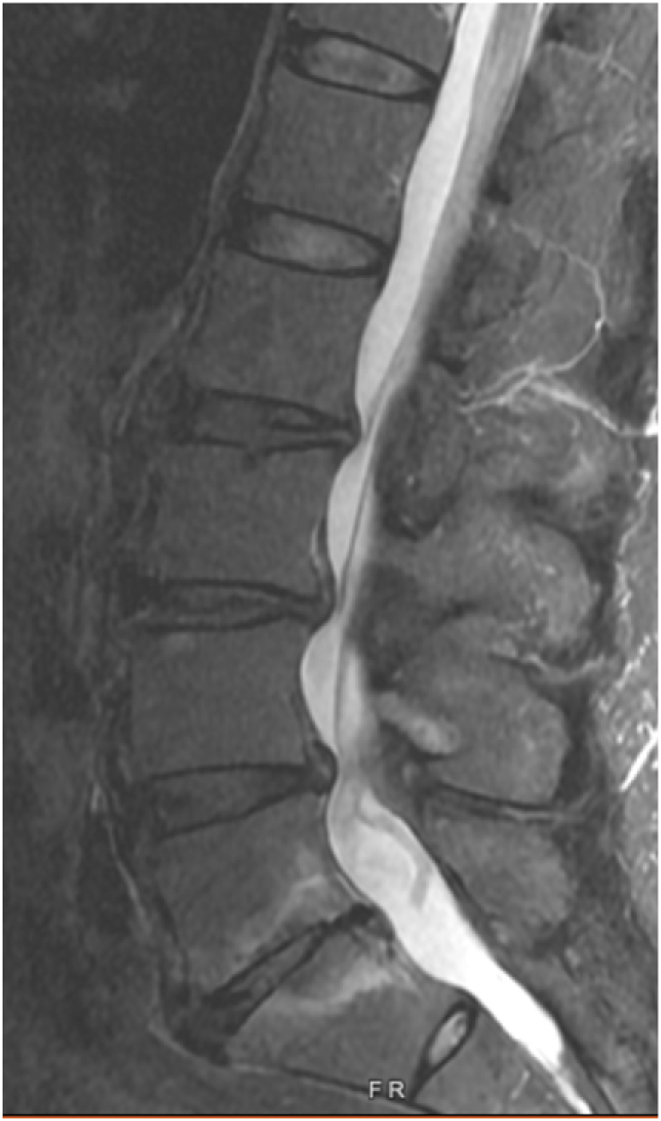
Sagittal STIR MRI pre-procedure.

**Figure 3B:**
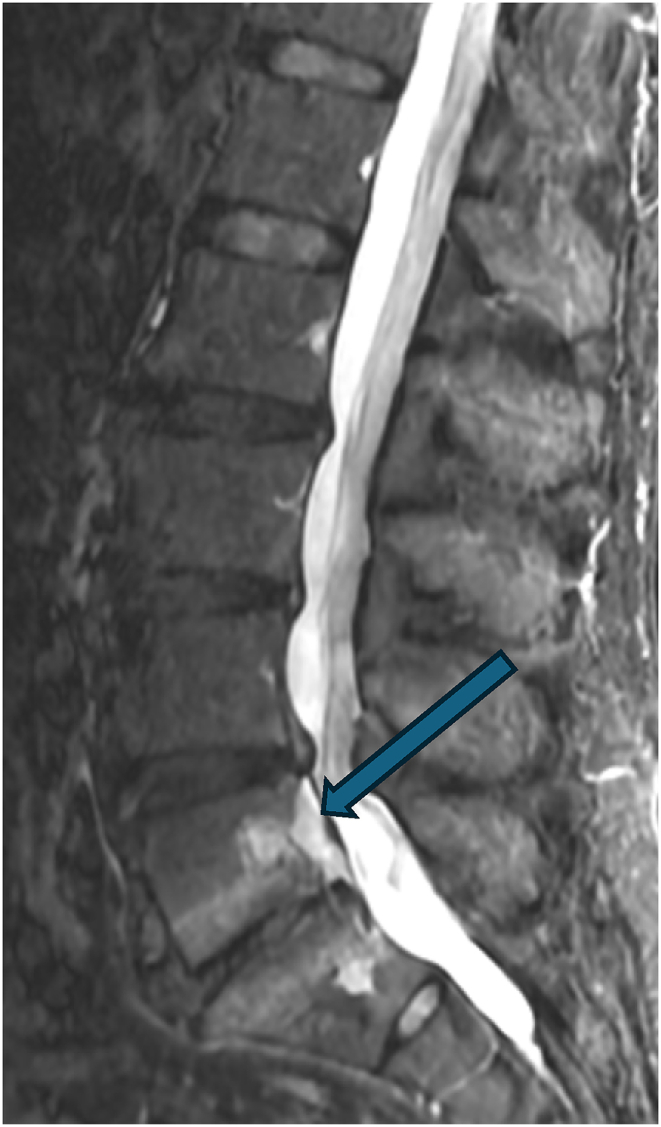
Sagittal STIR MRI 8 months post-procedure. Arrow demonstrates hematoma posterior to the L5 vertebral body.

**Fig. 3C:**
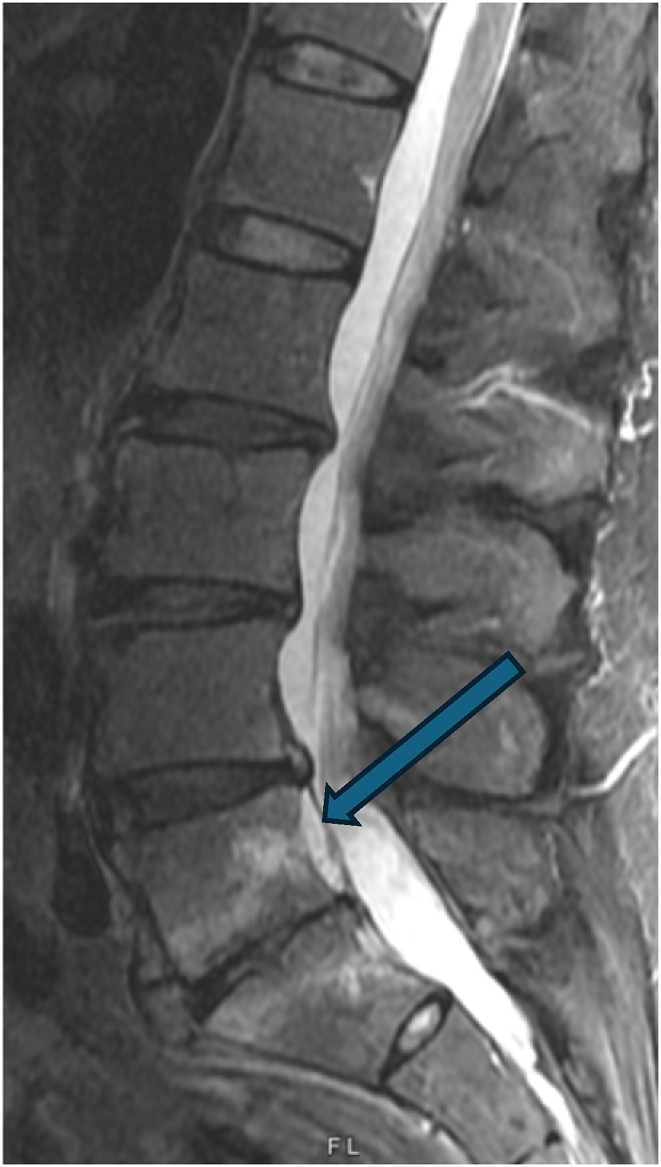
Sagittal STIR MRI 11 months post-procedure. Arrow demonstrates a smaller hematoma posterior to the L5 vertebral body.

Written informed consent was obtained from this patient to publish his case.

## 3 Discussion

**Fig. 4:**
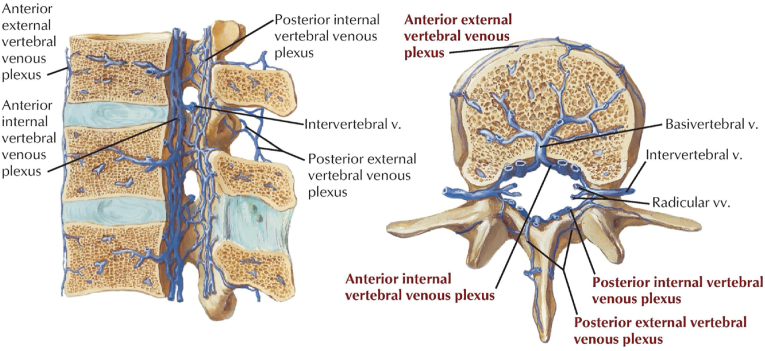
Basivertebral nerve draining into the anterior internal vertebral venous plexus. (From Hansen JT. Netter's Clinical Anatomy. 5th ed. Elsevier Health Sciences; 2022, with permission from Elsevier.) [6].

**Fig. 5:**
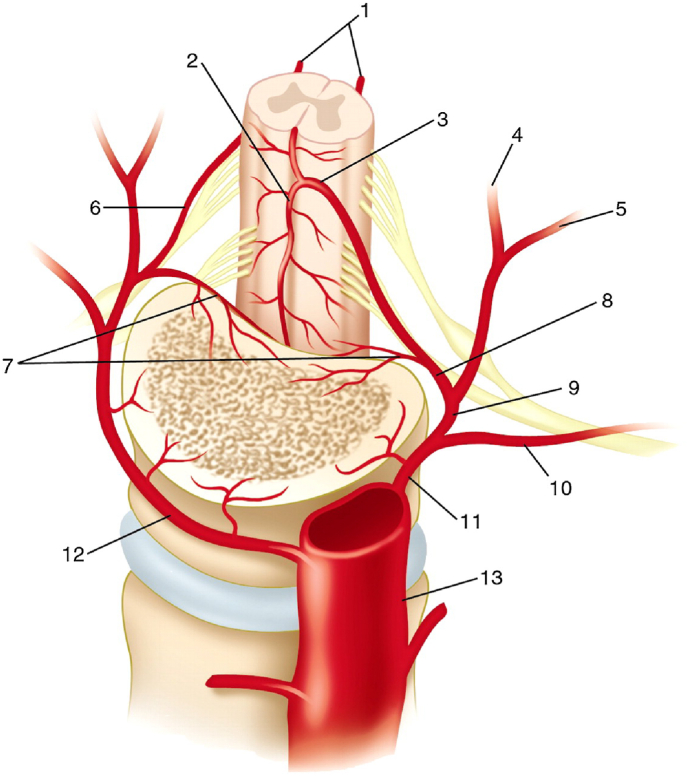
Arterial supply to the vertebral bodies from penetrating retrocorporeal arteries (7) branching from the segmental spinal arteries (8). Other vasculature: (1) posterior spinal arteries; (2) anterior spinal artery; (3) great anterior radiculomedullary artery or artery of Adamkiewicz; (4) medial musculocutaneous branch; (5) lateral musculocutaneous branch; (6) posterior radiculomedullary artery; (9) posterior (dorsal) branch; (10) anterior (ventral) branch; (11) left segmental artery (posterior intercostal artery); (12) right segmental artery (posterior intercostal artery); (13) aorta (From Alejandro Santillan et al. J Neurointervent Surg 2012; 4:67–74, with permission from BMJ Publishing Group Ltd.). [7].

This case brings attention to a theoretical risk that has yet to be observed with significant frequency in BVNA. Hematoma formation is plausible given the vasculature encountered in the procedure. Using the Intracept ® System protocol, an introducer cannula and stylet assembly are sequentially inserted through the pedicle into the vertebral body creating a channel directed at the basivertebral nerve terminus. The radiofrequency probe is then passed through this channel and heated to create a lesion at the target site. In this sequence, the cannula, stylet, and radiofrequency probe pass directly through the basivertebral foramen, which houses not only the basivertebral nerve, but also the basivertebral vessels. The basivertebral veins travel alongside the basivertebral nerves and converge to drain into the anterior internal venous plexus near the basivertebral nerve terminus (Fig. 4). The arterial supply appears to be less vulnerable from this procedure (Fig. 5).

In this case, the patient's pain had improved by 90% two weeks post-procedure but progressively returned to pre-procedure baseline over several months. Given the substantial initial improvement and steady decline over the next several months, it appears likely that the subsequently discovered hematoma was responsible for the eventual treatment failure. The partial resolution of the hematoma on follow-up MRI corresponding to the patient's partial improvement in low back pain further supports this hypothesis.

The timing of hematoma formation in relation to this patient's worsening symptoms is not entirely clear. We suspect that the hematoma occurred soon after the procedure and expanded over time, eventually distending the posterior longitudinal ligament and resulting in pain. However, we would have expected this process to occur more rapidly than it did. It is possible that the posterior longitudinal ligament limited expansion of the bleed which therefore formed more slowly, as opposed to what would be expected from the formation of a retroperitoneal hematoma which would potentially cause more acute symptoms related to hemodynamic compromise. Retroperitoneal hemorrhage has been documented on one occasion after BVNA due to an excessively lateral position of the trocar, with subsequent transient neuropraxia of the femoral nerve [2]. It is also possible that the patient's Parkinson's disease affected his ability to communicate his worsening pain, and that it may have occurred earlier than he reported it. Alternatively, a spontaneous hematoma formation, unrelated to the procedure, is another possible explanation. Spontaneous epidural hematoma formation is very rare, with estimates at 0.1 in 100,000 patients per year [8]. However, we are unaware of any reports of spontaneous hematoma formation in the location where it occurred in this patient, referred to as the extradural neural axis compartment (posterior to the vertebral body, anterior to the posterior longitudinal ligament) [9]. Therefore, we do suspect that the hematoma formation was procedural in origin due to bleeding from the basivertebral vein.

While this finding appears to be rare based on prior studies, it is an important consideration as hematoma formation could potentially lead to neurologic deficits and account for treatment failures. In patients who experience increased low back pain following a BVNA, post-procedure hematoma should be considered. In order to minimize this risk, close screening should be performed prior to BVNA with stringent criteria for holding anticoagulant medications, especially in patients with higher risk of bleeding.

## Declaration of competing interest

The authors declare that they have no known competing financial interests or personal relationships that could have appeared to influence the work reported in this paper.
